# Une forme pseudotumorale de cholécystite: la cholécystite xantogranulomateuse

**DOI:** 10.11604/pamj.2015.21.249.7603

**Published:** 2015-08-06

**Authors:** Ammar Mahmoudi, Abdelaziz Hamdi

**Affiliations:** 1 Service de Chirurgie Générale et Digestive, CHU Fattouma Bourguiba de Monastir, Monastir, Tunisie

**Keywords:** Cholécystite xantogranulomateuse, échographie, tomodensitométrie, chirurgie, anatomo-pathologie, xanthogranulomatous cholecystitis, ultrasound, CT scan, surgery, pathology

## Image en medicine

La cholécystite xantogranulomateuse est une forme particulière de cholécystite chronique, qui peut simuler une lésion tumorale aussi bien cliniquement, radiologiquement que macroscopiquement. La clinique n'est pas spécifique. Sa physiopathologie exacte n'est pas encore bien élucidée. Sa survenue peut cependant être expliquée par l'infection chronique, liée le plus souvent à des calculs, avec défaut d’évacuation de la bile, entraînant une extravasation de la bile dans la paroi conduisant à une accumulation d'histiocytes et au développement de micro-abcès qui seront remplacés, dans un second temps, par les nodules xanthogranulomateux. Une réaction fibreuse localisée peut également se voir expliquant les difficultés de diagnostic différentiel avec une tumeur. La connaissance du diagnostic en pré-opératoire permet d’éviter une chirurgie disproportionnée. Nous rapportons l'observation d'une femme de 62 ans, hypertendue, qui présentait des coliques hépatiques depuis deux ans. L'examen clinique était normal. L’échographie a montré une vésicule biliaire lithiasique à contenu hétérogène, à paroi très épaissie à plus de 12 mm, irrégulière, et siège de lésions nodulaires hypo-échogènes. La tomodensitométrie a trouvé une vésicule biliaire à contenu multilithiasique hétérogène, à paroi très épaissie et irrégulière, et prenant le contraste de façon hétérogène. Nous avons alors évoqué le diagnostic de carcinome vésiculaire. L'intervention chirurgicale, par laparotomie sous-costale droite, a montré une vésicule biliaire à paroi épaissie avec une fistulisation de microabcès visibles dans la lumière vésiculaire. L'examen histologique extemporané et définitif de la pièce de cholécystectomie était en faveur d'une cholécystite xanthogranulomateuse. L’évolution était sans complications avec un recul de six mois.

**Figure 1:**
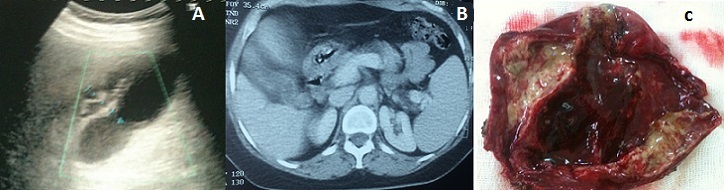
(A) échographie abdominale montrant une vésicule biliaire lithiasique à contenu hétérogène, à paroi très épaissie à plus de 12 mm, irrégulière, et siège de lésions nodulaires hypo-échogènes; (B) Tomodensitométrie abdominale montrant une vésicule biliaire à contenu multilithiasique hétérogène, et à paroi très épaissie et irrégulière; (C) Pièce opératoire de cholécystectomie ouverte montrant une vésicule biliaire à paroi très épaissie inflammatoire avec une fistulisation de microabcès visibles dans la lumière vésiculaire

